# Co-production as the ultimate goal; an incentive or discouragement?

**DOI:** 10.1186/s40900-025-00812-1

**Published:** 2025-12-22

**Authors:** A. Bangiri, A. Horobin, J. Baker, S. Pszczolkowski, S. Thust, P. S. Morgan

**Affiliations:** 1 University of Nottingham, Nottingham, UK; 2 Nottingham University Hospitals NHS Trust, Nottingham, UK; 3 Patient and Public Involvement Contributor, Doncaster, UK; 4 Nottingham NIHR Biomedical Research Centre, University of Nottingham, Nottingham, UK

**Keywords:** Patient and public involvement, PPI, Stereotactic radiosurgery, SRS, Cognitive decline, Quality of life questionnaire

## Abstract

Patient and public involvement (PPI) in research is a requirement in most of the western world. Funders routinely require extensive PPI plans from applicants and co-production is seen as the ultimate goal. The ladder of participation is unidimensional and does not account for the circumstances of the patients and their family members. Patients, especially those suffering from cancer, can be vulnerable and frail. Their disease and its unpredictable progression might prevent them from deep and lengthy involvement in research projects. Flexibility and adaptability is required from the research team to ensure that patients’ are involved to the level they want and are able to. Consequently, co-production might not be feasible in certain cases. The impact of the involvement of contributors should be regarded in its own merit, taking into account their circumstances, even if co-production is not achieved.

## Abstract (plain language summary)

When applying for funds, the research team has to prepare detailed plans about involving patients and the public throughout the project. Co-production is the gold standard. In co-production patients and family members with lived experiences as well as the public share the power to make decisions about the research and are recognised as equals. However, this might not always be possible. Cancer patients, are one example, where deep and prolonged participation cannot be achieved. They can be frail and vulnerable and their circumstances need to be a factor when making plans for involvement. Meaningful changes can be made to research plans even if co-production is not achieved.

## Introduction

Patient and Public Involvement (PPI) is clearly defined in the UK by the National Institute for Health Research (NIHR) and has become an integral part of health-related research [1]. The equivalent research infrastructures and national frameworks in Canada and the United States are the Patient Engagement Framework (SPOR) and the Patient-Centered Outcomes Research Institute (PCORI) respectively [2, 3]. In Australia, the National Framework for Consumer Involvement in Cancer Control covers the same principles with regards to cancer patients [4]. Involvement is about moving away from research being ‘imposed’ on patients and public without their input. This has, and continues to involve, a shift in academic culture towards collaboration, decision-sharing and deferring to members of the public.

In our recent publication, we detail how we involved frail and vulnerable brain tumour patients in the development of the protocol of the **Co**gnitive **De**cline after **B**rain **Rad**iosurgery (CoDe-B-Rad) study [5]. The CoDe-B-Rad study aims to identify new locations in the brain, that after treatment, might affect patients’ cognition and consequently quality of life. Patients recruited, receive a highly focused type of radiotherapy, and the study will look at potential short and long-term side-effects from the treatment.

Publications discussing the PPI aspects of projects, clinical trials and research in general have been steadily increasing [6–8]. Colomer-Lahiguera et al. showed that the numbers of publications reporting PPI in cancer research have doubled between 2018 and 2021, with the UK being the country where most publications originate [7]. In the UK, major public and charitable research funders require applications to include extensive PPI plans. The National Institute for Health and Care Research (NIHR) has published guidelines for researchers on how to approach PPI and how to compensate contributors [9–11]. They also published guidance on co-production and view this approach as underpinning all their involvement efforts going forwards through their Strategic Commitments for Public Partnerships. Key principles listed in this guidance include sharing of power, where there is joint ownership of key decisions [12, 13].

Sherry Arnstein’s Ladder of Participation from 1969 was seminal in highlighting how public institutions deny power to citizens. She described a typology of citizen participation presented as a ladder, with each ascending rung representing increasing levels of citizen control. It was a deliberately provocative challenge to the paternalistic status quo and has influenced later models of citizen participation [14].

Indigo Daya published the ‘participation ladder’ as a framework to more reproducibly categorise PPI activities in research [5]. Molony-Oates adapted Daya’s participation ladder and applied it to patient and public involvement in research in the UK [6]. Emphasis is placed on the outcome of where the balance of power lies in the decision-making process. It is implied that researchers increasingly recognise the value and contribution of PPI as you move up the ladder. The real-world context encompassing the research topic and the position of members of the public experiencing that context are not considered [14].

The aim of this commentary is to discuss the real world implications of involving frail and vulnerable populations in research, and that the value of patient input which sits on the lower rungs of the participation ladder, should not be underestimated.

## Reflections

Critics of Arnstein’s ladder exist [15, 16]. They posit that the exclusive focus on where power/the decision-making lies, is insufficient to fully appreciate the nature of participation [15, 16]. Whilst involving patients, carers and members of the public, where they have genuine power to make decisions in research, is vital to progressing PPI, the perception that achieving less than that, is somehow deficient, could be discouraging to researchers. Especially researchers who want to involve very vulnerable patient groups who may find a deep level of involvement too burdensome. For example, Ludwig et al. identified continuity of involvement as a major perceived barrier, noting researchers’ concerns that partnering with frail or seriously ill patients might be compromised by health deterioration [17]. Conversely, it could encourage researchers to think more inventively around how to better partner with patients and public in a meaningful and sympathetic way.

Other frameworks have been proposed to help increase and support patient and public participation in research [18, 19], These demonstrate better the dynamic aspect of PPI and that deeper involvement is not always feasible [18, 19], A detailed systematic review was published in 2019 by Greenhalg et al. detailing the different PPI frameworks [20]. Frameworks were categorised into five groups: power-focused, priority setting, study focused, report focused and partnership focused. Despite the fact that more than 60 frameworks exist, Greenhalg et al. found that the majority of these were only used within the group that developed them [20]. It appears that the ladder of participation remains one of the most commonly used frameworks, despite its obvious limitations.

Cancer patients can be an extremely vulnerable population. They can remain seriously ill over time or move into survivorship with long-term debilitating side-effects [7, 8] and the psychological burden of a relapse risk. Care should be taken to meet the specific needs and preferences of these patients and their carers when planning PPI activities [9] as there can be a great emotional and physical strain for both patients and carers alike. Hubbard et al. showed that involving people in research who have been affected by cancer, directly or indirectly, has enriched research design, improved recruitment as well as response rates. Specifically, this involvement allowed researchers to interact with groups that would otherwise be underrepresented [8]. Bergin et al. in their systematic review of PPI in cancer research found that the positive impacts of PPI led to improved study designs, more sustained recruitment, and a higher chance of clinical implementation of research findings amongst other advantages [21].

In our previous work, we involved cancer patients with brain tumours. Patients who expressed an interest to contribute, while willing to commit to meetings on a pre-determined day as a group, unfortunately could not carry through with their intentions [5]. Likewise, attending a series of meetings, or inputting on a frequent basis as project team members, in a more co-productive relationship, may not have been compatible with their situation.

The research team was flexible and adaptable, and by changing the original PPI plans, managed to include patients and carers with lived experiences in the protocol development for the CoDe-B-Rad study. Originally, group discussions were planned, but these evolved to one-to-one discussions, and online questionnaires. Group discussions were held with a regional patient support group that was already in place within the East Midlands. As a result of the consultations the focus of the study was steered towards the physical and mental symptoms of brain radiotherapy including cognitive decline. Quality of life questionnaires were included, as contributors advised that QoL was highly affected after treatment. Finally, the total testing time was restricted to 30 min, instead of the initial 60 allowed by the research team. Testing would be offered, face-to-face and online, in hospital or in the patient’s home to provide flexibility and minimise the burden on future participants, as advised by contributors. Finally, the Patient information sheets were amended, so as to make them more accessible and a mental health support pack was created in case any of the study participants became distressed [5].

Co-production is often seen as a goal for PPI in healthcare research. However, this needs to be tailored to the needs of the contributors. The research team involved patients and carers at the level of ‘Consult’ on the adaption of the participation ladder [22]. The ‘Consult’ type of participation is defined as ‘Researchers ask people with lived experience what they think and this becomes one of many considerations. This is commonly achieved by holding dedicated consultation sessions or surveys with people.’ The implications are that research is done for people, people are without power and have limited opportunities to have their say. It is stated that at this level, ‘Researchers hold the power and value their own expertise most of all’ [22].

On the contrary, our team valued the contributors’ input, as evidenced by the extent to which the research protocol was built around their comments and suggestions [5]. Additionally, the team ensured that feedback was provided and found ways to thank them for their input [5]. Moreover, the research team sought to address the power imbalance through attending the contributors’ support group session – developing a mutually positive relationship – and by communicating opportunities through brain tumour and cancer charities who are trusted sources of support for patients and carers. While contributors did not share in the decision-making, as they were not present or included when decisions about shaping the research were made, there was considerable shared learning – an aspect which does not feature in the participation ladder model approach.

Marcu et al. explored how PPI could be facilitated in mesothelioma research [23]. Similar to the brain tumour patients we involved, mesothelioma patients have high symptom burden and a poor prognosis. They found that both researchers and patients alike, highlighted this as a barrier to sustained involvement [23]. The team concludes that researchers should understand different approaches to PPI, ranging from consultation to co-design (and co-production), to be able to choose the most appropriate and time-effective method of involvement prior to including patients, carers and members of the public in mesothelioma research [23]. We concur with this approach for involving vulnerable patient groups. Being mindful of patient and carer needs whilst remaining open to deeper involvement, should a patient or carer feel able and willing, is key.

Wright et al. published recommendations on how to involve cancer patients receiving end of life care [24]. They highlighted the importance of being adaptable and flexible to new ways of working, similar to our findings [5, 24].

## Conclusion

Although co-production is seen as the gold standard in PPI, meaningful contributions and valuable shared learning can happen in the lower steps of the ‘ladder’. These can also shape research around the needs of patients. The challenges that vulnerable and frail patient populations, such as cancer patients, might face should always be considered. Occasionally, co-design/co-production might not be achievable, and contributions at the other steps of the ladder should not be seen as ‘less than’, merely for that reason. The impact remains; research is shaped around the needs of patients.

## Data Availability

No data are available with this submission. For data related to the original article please see the supplementary information of the Bangiri A et al. publication.
